# Did health reform improve financial protection for disadvantaged groups in Ecuador? A socio-economic inequality assessment of catastrophic health expenditures 2006-2014

**DOI:** 10.1136/bmjopen-2025-100522

**Published:** 2025-07-30

**Authors:** Edy Quizhpe Ordóñez, Miguel San Sebastian, Enrique Teran, Anni-Maria Pulkki-Brännström

**Affiliations:** 1 Department of Epidemiology and Global Health, Umeå University, Umeå, Sweden; 2 Colegio Ciencias de la Salud, Quito, Ecuador

**Keywords:** Health, Cross-Sectional Studies, Public Health

## Abstract

**Abstract:**

**Objectives:**

Latin American countries have long struggled with socioeconomic inequalities and health equity. In 2007, Ecuador implemented a health reform to address these issues by making public health services free, coordinating finances between subsystems and increasing the state’s health budget. This study evaluates whether Ecuador’s health system reform (2007–2017) reduced out-of-pocket (OOP) health spending, catastrophic health spending (CHS) and socioeconomic inequalities in CHS.

**Design:**

Cross-sectional study.

**Setting:**

Secondary data available of households from the 2006 and 2014 National Living Standards Measurement surveys.

**Methods:**

Descriptive statistics (means and medians) and log-binomial regression were applied to assess prevalence of OOP and socioeconomic inequalities (residence, region, health insurance status and wealth) in catastrophic health expenditure (CHE) for each period and over time.

**Results:**

Overall, there was a significant reduction of 14% points in the proportion of households with OOP healthcare expenditure. The prevalence of CHE decreased from 17% to 10% and within each socioeconomic group over time. Significant reductions in relative risk were observed in all socioeconomic variables. The inequality in CHE decreased significantly in households placed in rural areas (relative difference (RD): 0.88; 95% CI: 0.79 to 0.97) and poorest (RD: 0.82; 95% CI: 0.69 to 0.97); however, it increased within regions (RD: 0.58; 95% CI: 0.44 to 0.76) and for uninsured households (RD: 1.39; 95% CI: 0.95 to 2.04).

**Conclusions:**

This study suggests that recent health reform effectively reduced OOP healthcare expenditure, CHE and some socioeconomic inequalities. Future reforms should further invest in key areas, expand health insurance for the most disadvantaged and monitor progress towards universal health coverage to address persistent inequalities.

**STRENGTHS AND LIMITATIONS OF THIS STUDY**

- The analysis includes nationally representative data covering diverse socioeconomic groups, with information collected directly from households in Ecuador.
- Provides an assessment of the impact of health reforms to achieve universal health coverage.
- Response and recall bias may be present due to the questionnaire’s length and the complexity or sensitivity of some questions.
- Although the healthcare reform was implemented between 2007 and 2017, this study only included data to evaluate the effects of the reform from two points in time (2006 and 2014).
- Due to the structure of the data sets, it was not possible to use other relevant data in this analysis such as household size, the age of the household members and the education level of the household head.

## Introduction

 It is essential that any health system includes a robust financial protection component. In conjunction with delivering quality health services that are responsive to the needs of the population, this serves to protect individuals from financial hardship. The Universal Health Coverage (UHC) strategy, as advocated by the WHO, aims to reinforce and establish health financing policies with the objective of reducing catastrophic expenditure and household impoverishment. Most of these policies entail an increase in the allocation of financial resources in the public health budget, the pooling of resources among public providers and improvements in the purchasing and payment processes for essential health services between public and private actors.^1 2^

Despite the implementation of numerous reforms in several Latin American countries with the objective of achieving UHC, significant challenges remain, particularly in terms of meeting the needs of marginalised groups and reducing socioeconomic inequalities in health outcomes.^3 4^

A review of the literature from studies conducted in Latin America and the Caribbean (LAC) revealed a considerable degree of inequality in out-of-pocket (OOP) expenditure and catastrophic health expenditure (CHE) between countries.^5 6^ Similarly, a study of 12 LAC countries conducted in 2006 found CHE rates ranging from 1% to 25%, including data from Ecuador.^7^ While CHE commonly affects households with low incomes, as well as those living in rural areas and the uninsured,^8^,^10^ the Mexican experience demonstrates that appropriate health system reforms can reduce social inequalities in CHE.^11^

### Study context

Historically, Ecuador’s health system has been characterised by significant fragmentation and segmentation.^12^ Prior to the reform, the Ministry of Public Health (MoPH) was the primary provider of public health services, which covered approximately 60% of the population. Most of this population was self-employed or unemployed and belonged to middle or low-income families. Except for maternal and child healthcare, these services were typically paid OOP directly at the point of care. Other groups, including the armed forces, the police, voluntary contributors, pensioners, public and private civil servants, and workers in formal employment, comprising approximately 30% of the population, had access to healthcare as part of social health insurance benefits through a prepaid model included in their social security schemes. The remaining 10% of the population was covered by the private healthcare sector.^12^ The MoPH receives financial resources from the Ministry of Finance based on historical assignments and mainly from taxes, oil revenues and external funds. Subsequently, the funds are distributed to administrative units and hospital health facilities throughout the country. The social security systems are financed through compulsory payroll taxes and voluntary user contributions, with the provision of services conducted through the utilisation of their own facilities. The MoPH accounts for over half of the total health expenses in the public system. In the private sector, health insurance companies operate prepaid drug schemes financed by beneficiary contributions. During this period, approximately 27% of health needs remained unmet due to limited access to services.^13 14^

In 2007, the newly elected government initiated a series of significant social and health reforms with the objective of enhancing the quality of life of the population.^15^ The reforms were designed to extend coverage and accessibility to quality health services across the public sector through an improvement in the financing and capacity building.^16^ In 2008, the Constitution was amended to recognise health as a fundamental human right, guaranteed by the state. The government then integrated the reforms into a national development plan to achieve the stated goals.^17^

The objective of the health financing reforms was to reduce both direct health expenditure and CHE. Several strategies were implemented, beginning with the decision to make all existing MoPH health services available free of charge. This was designed to increase utilisation of health services among the most disadvantaged sections of the population, with the full cost of these services being covered and no copayments required. Subsequently, additional health services were incorporated into the comprehensive health packages, aligned with the life stages of the target population, with the objective of optimising health benefits. Second, financial resources were mobilised from the national accounts, thereby increasing the fiscal space for the health budget on an annual basis. Thus, the current expenditure on health increased from 10.54% to 12.36% of gross domestic product in 2017.^18^ Third, the constitution established a Comprehensive Public Health Network (Red Pública Integral de Salud—RPIS in Spanish), led by the MoPH, with the objective of ensuring continuity of care and reducing the structural fragmentation of the system. Ultimately, the Ecuadorian Social Security Institute (Instituto Ecuatoriano de Seguridad Social—ie, SS in Spanish) witnessed a significant expansion in its coverage, rising from 26% in 2007 to 43% in 2013.^19 20^

These processes increased medical consultations across all care levels. The extra budget was mainly used for infrastructure, supplies, pharmaceuticals and hiring healthcare professionals.^21^ A total of 5000 new health workers were recruited, primarily for positions in community and hospital settings. Furthermore, 47 new and upgraded hospitals and 73 first-level health centres were incorporated into the public health network. The establishment of this network also prompted the MoPH to redesign the comprehensive healthcare model, with the aim of facilitating patient referrals between public and private providers. This had the potential to reduce healthcare costs and delays in care. An official agreement implemented by the MoPH established a national cost tariff and reimbursement and payment mechanisms through fee-for-service.^22 23^

Some studies have indicated that the reform enhanced access for the general population and reduced socioeconomic inequalities in healthcare utilisation among the most vulnerable groups.^24 25^ However, there is a paucity of literature evaluating the impact of the reform in terms of financial protection. Consequently, this study aimed to assess the impact of the health reform on (1) reducing OOP health expenditure and CHE and (2) decreasing socioeconomic inequalities in CHE during the period 2006–2014.

## Methods

This was a cross-sectional study that used publicly available secondary data from the National Living Standards Measurement surveys. These were conducted by the National Institute of Statistics and Censuses (Instituto Nacional de Estadísticas y Censos in Spanish) in 2006 (pre-reform) and 2014 (during the reform) across the country.^26^ The surveys provided nationally representative population information from 13 581 and 28 970 households in each wave respectively. In each wave, the sampling method was multistage, stratified and proportional to the size of the population. The households were randomly selected from a complete and updated list of occupied households, with the same probability of selection being applied to each house. Additionally, the population was stratified by region and by rural or urban residence. The units initially sampled, the Primary Sampling Units, were clustered census sectors, while the households constituted the Secondary Sampling Units.^26^ Properly trained local interviewers oversaw and collected the information from each dwelling, making the necessary visits to complete the survey information. The time taken to complete the surveys was around 1 year in each period.

The questionnaire comprised several modules. In this study, we only used the information at the household level, which included household expenditure on health-related items (including drugs, consultations, hospitalisation and other services), other subsistence and non-subsistence expenditure, family assets, the status of health insurance coverage and a range of sociodemographic characteristics. The individual within the household with the most comprehensive understanding of the expenditure was responsible for providing the data on behalf of the family to the trained interviewers in each survey wave. Ethical approval and consent were not required for this study because the data were publicly available and came from secondary sources. Overall, the amount of missing data was minimal, with less than 1% in the wealth index and none in the other social variables and outcomes. As a result, no methods were implemented to address missing data.

### Variables

This study used two indicators of financial protection: out-of-pocket health expenditure (OOPhe) and CHE. OOPhe was defined as any expenditure on health services by any member of the household in the previous 3 months. This included direct costs of general and prenatal care, including physician fees, medicines, hospitalisation, laboratory and imaging services, and alternative/traditional medicine. As in other studies, indirect costs such as waiting time for services, transport to the point of care, parenteral/enteral nutrition formulas and health insurance premiums were excluded.^27^ All costs were then aggregated into a monthly average representing the household’s total OOPhe and categorised separately into general healthcare and pregnancy/childbirth care.

The CHE was constructed in two steps. First, the household’s capacity to pay (CTP) was calculated. CTP was defined as the household’s non-food expenditure in a given period and included four types of expenditure: (1) expenditure on fuel and energy in the last week; (2) expenditure on household and personal care products, entertainment, culture, membership fees and domestic services in the last month; (3) expenditure on clothing and footwear in the last 3 months and (4) expenditure on appliances, furniture, leisure, sports equipment, jewellery, vehicles, transport, professional services, prepaid fees, taxes and transfers to family members in the last year. Second, CHE was determined using the methodology proposed by WHO in 2004, where households were exposed to CHE if their OOP health expenditure was equal to or greater than 40% of their CTP.^28 29^ This approach allowed us to compare our findings with data from previous studies conducted nationally and from neighbouring countries in the region.

Four socioeconomic variables or social stratifiers were selected from the data set. Geographical variables included the Ecuadorian regions (Andean, Coast and Amazon) and place of residence (rural and urban). A household wealth index was derived using principal component analysis of 15 household assets reported in the surveys including doorway materials, roof, wall and floor material of household, household construction, cooking facility, cooking fuel, toilet, water source, lighting source, landline telephone, internet at home, satellite TV and household waste disposal. The PCA was run separately for 2006 and 2014, and the index was divided into quintiles, with the first quintile as the richest.^30^ Finally, health insurance status was obtained if at least one family member had a public or private health insurance.

### Data analysis

For the first objective, we calculated the proportion of households with OOP health expenditure and the mean and median monthly expenditure for the two periods, 2006 and 2014. To facilitate comparison over time, the 2006 values were adjusted using the 2014 Consumer Price Index, multiplied by a factor of 1.41.^31^ All expenditures were measured in US dollars ($), the current currency in Ecuador.

For the CHE, all expenditures were aggregated into a monthly average, which was then divided by the CTP to obtain the household’s OOP health expenditures (hOOPhe) as a share of the household’s CTP (hCTP). We then created a dummy variable where a value of 1 indicated a household with CHE if hOOPhe/hCTP≥0.4, and a value of 0 indicated no CHE if hOOPhe/hCTP<0.4. In addition, we calculated CHE with a threshold of 20% to allow comparison with other settings (online supplemental appendix 1).

For the second objective, a log-binomial regression model was used to calculate the relative risk (RR) of CHE for each socioeconomic variable in 2006 and 2014, highlighting differences between groups, also using 95% CI for statistical inference. First, a univariable model (model 1) was estimated, followed by a multivariable model (model 2) including all statistically significant variables from model 1. Finally, an interaction term was added to assess RR changes over time, expressed as relative differences (RD). RD less than one implies a reduction in RR over time compared with the reference group and thus a decrease in inequality. All analyses were performed using Stata V.15.1.

### Patient and public involvement statement

None.

## Results

**Figure 1:**
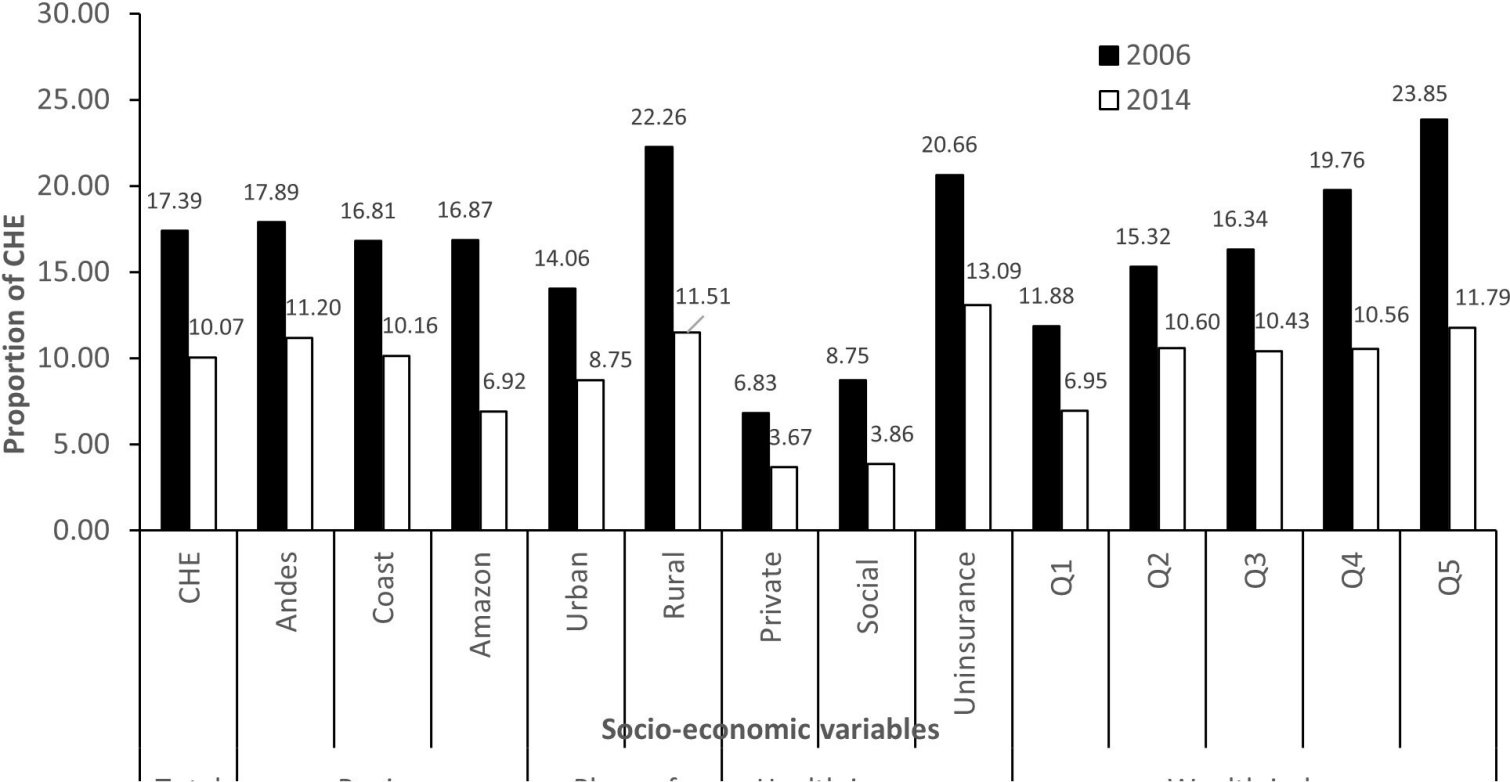
Prevalence of catastrophic health expenditure (CHE) by socioeconomic variable in 2006 and 2014. The X-axis represents the socioeconomic variables (region, place of residence, health insurance and household wealth index), and the Y-axis represents the percentage of catastrophic health expenditures. Data were obtained from the Living Conditions Survey conducted by the National Institute of Statistics and Census of Ecuador in 2006 (black bar) and 2014 (white bar).

Table 1 and figure 1 show descriptive statistics. In 2006, 89% of households reported direct health expenditure in the last 3 months, which decreased to 74% in 2014. The mean and median monthly expenditure decreased by 35% and 50%, respectively, with a greater reduction in general healthcare compared with pregnancy and delivery services. The most significant reductions occurred in spending on medicines and consultations with medical professionals (data not shown).

**Table 1:** Households with out-of-pocket health expenditure and the monthly average expenditure in 2006 and 2014

	2006				2014				Percentage change from 2006 to 2014		
Health expenses	Frequency	%	Mean ($)*	Median ($)*	Frequency	%	Mean ($)	Median ($)	Frequency % points	Mean %	Median %
Total	12 100	89.10	45.56	15.27	21 523	74.29	29.47	7.67	−14.4	−35.32	−49.77
Expenses for general healthcare	12 024	88.54	42.72	14.10	21 256	73.37	27.33	6.67	−15.17	−36.03	−52.70
Expenses for pregnancy and delivery	766	5.64	8.51	0.00	1237	4.27	6.42	0.00	−1.37	−24.56	

* Adjusted for inflation, 2014 values.

Figure 1 shows the proportion of households experiencing CHE in each socioeconomic group in the two periods. The overall prevalence of CHE decreased over time from 17% to 10%. In 2006, the proportion was highest among rural, uninsured and poorest households. In 2014, reductions were observed in all socioeconomic groups, with the largest reductions in the Amazon region (from 17% to 7%), rural areas (from 22% to 12%) and the poorest quintile (from 24% to 12%).

Table 2 shows the RR of CHE for different socioeconomic groups over time. In 2006, the multivariable model (model 2) showed statistically significant higher risks for rural areas (RR: 1.27) compared with urban areas, for uninsured households (RR: 2.57) compared with households with private health insurance and for all wealth quintiles compared with the richest quintile (RR: 1.65 in the poorest quintile), but no differences in risk by region. In 2014, a statistically significant lower risk of CHE was observed in the Amazon compared with the Andean region (RR: 0.59; 95% CI: 0.53 to 0.67). No differences in risk were observed by rurality or in wealth quintiles except for the second-richest quintile (RR: 1.24) in 2014. However, compared with households with private insurance, uninsured households still had a statistically significant higher risk of CHE (RR: 3.62) in 2014 while households with social security insurance had similar risk in both periods.

**Table 2:** Relative risk of catastrophic health expenditure by socioeconomic group in 2006 and 2014

		2006				2014				
		Model 1		Model 2			Model 1	Model 2		Interaction
Variables		RR	95% CI	95% CI		RR	95% CI	RR	95% CI	Relative differences 2014–2006
Region	Andean	1		1		1		1		
	Coast	0.93	(0.86 to 1.01)	0.94	(0.86 to 1.02)	0.90	(0.84 to 0.97)*	0.92	(0.85 to 1.00)	0.86 (0.78 to 0.96)*
	Amazon	0.94	(0.81 to 1.09)	0.87	(0.75 to 1.01)	0.61	(0.54 to 0.69)*	0.59	(0.53 to 0.67)*	0.58 (0.44 to 0.76)*
Place of residence	Urban	1		1		1		1		
	Rural	1.58	(1.47 to 1.70)*	1.27	(1.14 to 1.40)*	1.31	(1.22 to 1.40)*	1.05	(0.96 to 1.14)	0.88 (0.79 to 0.97)*
Health insurance	Private	1		1		1		1		
	Social security	1.28	(0.92 to 1.78)	1.22	(0.87 to 1.70)	1.05	(0.72 to 1.51)	1.07	(0.74 to 1.55)	0.99 (0.66 to 1.48)
	Uninsured	3.02	(2.21 to 4.14)*	2.57	(1.87 to 3.53)*	3.56	(2.50 to 5.07)*	3.62	(2.53 to 5.17)*	1.39 (0.95 to 2.04)
Wealth index	Q1 (richest)	1		1		1		1		
	Q2	1.28	(1.12 to 1.47)*	1.27	(1.11 to 1.46)*	1.52	(1.34 to 1.72)*	1.24	(1.09 to 1.39)*	1.14 (0.97 to 1.35)
	Q3	1.37	(1.20 to 1.57)*	1.30	(1.13 to 1.49)*	1.50	(1.32 to 1.69)*	1.08	(0.95 to 1.22)	1.15 (0.97 to 1.36)
	Q4	1.66	(1.46 to 1.89)*	1.42	(1.22 to 1.64)*	1.51	(1.34 to 1.71)*	0.99	(0.86 to 1.13)	0.95 (0.80 to 1.13)
	Q5 (poorest)	2.00	(1.77 to 2.27)*	1.65	(1.41 to 1.93)*	1.69	(1.50 to 1.90)*	1.06	(0.92 to 1.22)	0.82 (0.69 to 0.97)*

* Statistically significant.

RR, relative risk .

Statistically significant reductions in RR over time were observed in rural areas (RD: 0.88; 95% CI: 0.79 to 0.97) and among the poorest (RD: 0.82; 95% CI: 0.69 to 0.97). However, the risk increased in the Andean region (Coast RD: 0.86; 95% CI: 0.78 to 0.96, Amazon RD: 0.58; 95% CI: 0.44 to 0.76), and among uninsured households, although not significantly (RD: 1.39; 95% CI: 0.95 to 2.04). Observe that a decrease in RD implies an increase in inequality in the case of regions since the Andean was the reference group. The same trends were found for each social group when the 20% threshold was used (online supplemental table A1 in the annex).

## Discussion

This study evaluated the impact of the recent reform of the Ecuadorian health system on improving financial protection in healthcare. Our results showed a reduction in households’ direct healthcare expenditure and CHE during the reform compared with the prereform period. A significant reduction in socioeconomic inequalities in CHE was also observed between the richest and the poorest and between urban and rural households over time. However, inequalities in CHE increased for the Andean region and for uninsured households.

Several factors could explain the observed results. The significant increase in funding for the public health sector during the reform played a key role in reducing households’ OOP expenditure. In 2007, the government declared a state of emergency in the health sector and mobilised substantial resources to expand services and provide free healthcare at all levels of care, a commitment it maintained throughout its term. In addition, the better results in the Amazon and in rural areas could be explained by an increase in health insurance coverage in rural areas by the social security system for farmers and the expanded implementation of home visits through the integrated healthcare model in remote areas of the country.

These initiatives resulted in increased coverage of preventive and curative services, particularly in maternal and child health programmes and services for vulnerable groups such as the elderly, people with disabilities and low-income families. As a result, the OOP expenditure was reduced, particularly on essential medical procedures and medicines, which are the main drivers of health expenditure in the LAC region.^32 33^ Studies where countries have implemented similar policies in analogous contexts have also shown positive effects on health financial protection.^34 35^

Second, the renewed healthcare model established primary healthcare as the gateway to the rest of the healthcare system, providing a wide range of services, including cost-effective preventive services for chronic diseases, and strengthening the coordination of continuity of care. This financial investment reduced avoidable hospitalisations.^36^ In addition, the implementation of the comprehensive public health network included high-cost, life-saving interventions, such as kidney dialysis, cancer treatment, HIV/AIDS treatment and rare diseases in the health benefits free of charge. This may have contributed to reducing catastrophic expenditure. These results are similar to those found in other countries that have implemented universal healthcare policies.^37^

Third, numerous studies have shown that countries implementing health reforms with diverse health insurance systems can be effective in reducing catastrophic expenditure and impoverishment, especially for disadvantaged groups.^38^,^40^ Experience in similar contexts has shown that shifting from OOP health expenditure to pooling public health resources to provide a national health insurance has positive results in terms of coverage, especially for children, poor households and the elderly groups, who tend to have greater health needs and limited financial resources.^41^,^44^ However, even in countries such as Colombia, which has one of the highest rates of health insurance coverage, improving health outcomes remains a challenge, particularly in terms of equity.^45 46^

Finally, despite progress in health financing protection, greater reduction in OOPhe and CHE could have been expected, especially among uninsured households, since all healthcare services were free at all levels. Inefficiencies in the management of local health systems (hospitals and health districts) and in supply processes led, however, to continuous stock-outs of drugs and medical equipment in health facilities, forcing families to seek care at private pharmacies, a situation that persisted throughout most of the reform period.

## Conclusion

The recent reform in Ecuador has reduced both total OOP expenditure and CHE. It has also improved financial protection for the most disadvantaged groups, notably reducing socioeconomic inequalities in CHE over time, except in the Andean region and among uninsured households. Therefore, it is imperative to review and adapt the reforms implemented to expand and sustain universal access to healthcare and improve health insurance coverage for the most disadvantaged groups. A forward-looking approach to the financial sustainability of the public healthcare model is required to ensure the right to health in the country.

## Supplementary material

10.1136/bmjopen-2025-100522 online supplemental file 1

## Data Availability

Data are available in a public, open access repository.
