# Supplementary material for: Did health reform improve financial protection for disadvantaged groups in Ecuador? A socio-economic inequality assessment of catastrophic health expenditures 2006-2014
Source: BMJ Open. 2025 Jul 30;15(7):e100522. doi: 10.1136/bmjopen-2025-100522 (PMC12314978; doi:10.1136/bmjopen-2025-100522)
Supplement: online supplemental file 1 [file bmjopen-15-7-s001.docx]

**Annex**

**Table A1. Relative risk of catastrophic health expenditure by socio-economic group in 2006 and 2014. Threshold 20%**

|  |  |  | **2006** |  |  |  |  | **2014** |  |  |  |  |  |
| --- | --- | --- | --- | --- | --- | --- | --- | --- | --- | --- | --- | --- | --- |
|  |  | **Model 1** |  | **Model 2** |  |  | **Model 1** |  | **Model 2** |  |  |  | **Interaction** |
| **Variables** | | **RR** | **95% CI** | **RR** | **95% CI** |  | **RR** | **95% CI** | **RR** | **95% CI** | **Relative Differences**  **2014-2006** | | |
| Region | Andean | 1 |  | 1 |  |  | 1 |  | 1 |  |  | 0.94  0.73 | (0.88-1.00)  (0.61-0.86)* |
|  | Coast | 1.02 | (0.98-1.07) | 1.03 | (0.98-1.08) |  | 1.03 | (0.98-1.07) | 1.05 | (1.00-1.10) |  |  |  |
|  | Amazon | 0.91 | (0.83-1.00) | 0.90 | (0.83-0.99)* |  | 0.68 | (0.64-0.73)* | 0.68 | (0.64-0.74)* |  |  |  |
| Place of residence | Urban | 1 |  | 1 |  |  | 1 |  | 1 |  |  | 0.91 | (0.85-0.96)* |
|  | Rural | 1.23 | (1.17-1.28) | 1.08 | (1.02-1.14) |  | 1.07 | (1.03-1.12) | 0.97 | (0.92-1.02) |  |  |  |
| Health insurance | Private | 1 |  | 1 |  |  | 1 |  | 1 |  |  | 0.98  1.28 | (0.83-1.17)  (1.08-1.50)* |
|  | Social security | 0.95 | (0.82-1.10) | 0.92 | (0.80-1.07) |  | 0.97 | (0.82-1.15) | 1.02 | (0.86-1.22) |  |  |  |
|  | Without insurance | 1.42 | (1.24-1.63)* | 1.33 | (1.16-1.52)* |  | 1.84 | (1.56-2.16)* | 2.01 | (1.71-2.38)* |  |  |  |
| Wealth index | Q1 (richest) | 1 |  | 1 |  |  | 1 |  | 1 |  |  | 1.08  0.97  0.92  0.89 | (0.99-1.19)  (0.89-1.07)  (0.83-1.01)  (0.80-0.98)* |
|  | Q2 | 1.15 | (1.06-1.24)* | 1.07 | (0.99-1.15) |  | 1.25 | (1.16-1.33)* | 1.12 | (1.04-1.19)* |  |  |  |
|  | Q3 | 1.25 | (1.16-1.35)* | 1.11 | (1.03-1.20)* |  | 1.17 | (1.09-1.26)* | 0.98 | (0.91-1.05) |  |  |  |
|  | Q4 | 1.33 | (1.24-1.43)* | 1.10 | (1.01-1.20)* |  | 1.17 | (1.09-1.25)* | 0.93 | (0.86-1.01) |  |  |  |
|  | Q5 (poorest) | 1.39 | (1.29-1.49)* | 1.11 | (1.01-1.22)* |  | 1.17 | (1.09-1.26)* | 0.92 | (0.84-0.99)* |  |  |  |

*RR: relative risk*

** Statistically significant*
