# Supplementary material for: Did health reform improve financial protection for disadvantaged groups in Ecuador? A socio-economic inequality assessment of catastrophic health expenditures 2006-2014
Source: BMJ Open. 2025 Jul 30;15(7):e100522. doi: 10.1136/bmjopen-2025-100522 (PMC12314978; doi:10.1136/bmjopen-2025-100522)
Supplement: Supplementary file 2 [file bmjopen-2025-100522.reviewer_comments.pdf]

PEER REVIEW HISTORY

BMJ Open publishes all reviews undertaken for accepted manuscripts. Reviewers are asked to complete a checklist review form and are provided with free text boxes to elaborate on their assessment. These free text comments are reproduced below.

ARTICLE DETAILS

Title (Provisional)

Catastrophic healthcare expenditure in Ecuador: Impact of health reform on socio-economic inequalities – A before-and-after cross-sectional study

Authors

Quizhpe Ordóñez , Edy; San Sebastian, Miguel; Teran, Enrique; Pulkki-Brännström, Anni-Maria

---

VERSION 1 - REVIEW

**Reviewer**	**1**
**Name**	**Otieku, Evans**
**Affiliation**	**University of Ghana, Institute of Statistical, Social and Economic Research**
**Date**	**25-Mar-2025**
**COI**	**None**

Comments for the authors.

Abstract

The methods section should include all statistical methods used. For instance, how was mean and median values estimated. Once these estimates are accompanied by 95% CI it is no longer in the domain of descriptive statistics. This should be elaborated in the main methods as well.

Data and Statistical Analysis

The authors said they used a before and after cross-sectional design, in which data from 13,581 and 28,970 participants were collected, respectively. Is the data paired or unpaired (parametric or non-parametric)? For this kind of impact analysis, paired data will be ideal to evaluate changes attributable to the policy, but the sample in each group suggests unpaired data was used. Could the authors clarify that?

Having transformed the data on a log scale, did they check the assumption of normality and equal variance between groups before using the log-binomial regression? Any evidence of this assumption test in supplementary material will be helpful, as failure to check the assumption may lead to an inaccurate conclusion.

What statistical method did they use to calculate differences in the mean OOP between groups? Paired or unpaired t-test? Why present a median estimate? What statistical assumption informed that? Is the data parametric or non-parametric?

Why use CPI to adjust costs from local currency to USD? The standard practice is to adjust using Purchasing Power Parity equivalent in USD for comparison of cost across countries. https://eppi.ioe.ac.uk/costconversion/

Though 40% and 20% CHE thresholds are accurate, they could consider 15% and 25% standard thresholds. This will help understand the dynamics better as 40% is too high and may exclude several households and individuals who may experience impoverishment beyond, for example, at 15% threshold. Additional analysis showing catastrophic overshoot and impoverishment could have been more interesting as that speak volume to policymakers.

Results

Some confidence intervals accompanying the point estimate are inaccurate, as the margin of error is inconsistent when checked using the lower and upper uncertainty intervals. The authors should check this error in all Tables containing 95% CI.

Tables 2 and 1A: The presentation of wealth quintile groups may be misleading if the authors say Q1 is richest instead of poorest.

**Reviewer**	**2**
**Name**	**Rezaei, Satar**
**Affiliation**	**Kermanshah University of Medical Sciences, School of Public Health**
**Date**	**14-May-2025**
**COI**	**None**

Dear Editor

Thank you for giving me the opportunity to read and review this manuscript. I read it with great interest and believe the paper is suitable for publication in the journal after addressing all of my comments outlined below.

1- First of all, please revise the study title to better reflect the objectives. The current title is vague and misleading. I believe that a before-and-after cross-sectional study design is unnecessary.

2- In the study title, the phrase "health reform" appears, yet there is no information about health reforms in the abstract. Please either add more details about health reforms in the abstract or consider changing the study title accordingly.

3- Page 3, STRENGTHS AND LIMITATIONS OF THIS STUDY: The authors stated, “The method for calculating CHE could limit comparisons between countries in the region and globally.” I could not understand this limitation clearly. Please revise this statement for clarity or consider deleting it.

4- Page 3, the authors stated, “Although the healthcare reform was implemented between 2007 and 2017, the study only included data from two points in time (2006 and 2014), which are available at the moment.” It appears that the study did not compare the prevalence of CHE before and after the health reforms. Therefore, I suggest rephrasing or removing references to health reform throughout the paper, including the study title.

5- The introduction should be rewritten to focus more on studies conducted in other countries. Please incorporate the following references in both the introduction and discussion sections to enrich and improve the paper.

- Catastrophic health expenditure during the COVID-19 pandemic in five countries: a time-series analysis A. Haakenstad, C. Bintz, M. Knight, K. Bienhoff, H. Chacon-Torrico, W. H. Curioso, et al. The Lancet Global Health 2023 Vol. 11 Issue 10 Pages e1629-e1639 DOI: 10.1016/S2214-109X(23)00330-

- Al-Hanawi, M.K., Njagi, P. Assessing the inequality in out-of-pocket health expenditure among the chronically and non-chronically ill in Saudi Arabia: a Blinder-Oaxaca decomposition analysis. Int J Equity Health 21, 192 (2022). https://doi.org/10.1186/s12939-022-01810-5

- Rezaei S, Woldemichael A, Ebrahimi M, Ahmadi S. Trend and status of out-of-pocket payments for healthcare in Iran: equity and catastrophic effect. J Egypt Public Health Assoc. 2020 Nov 3;95(1):29. doi: 10.1186/s42506-020-00055-w. PMID: 33140214; PMCID: PMC7606373.

- Diego Proaño Falconi & Eduardo Bernabé, 2018. "Determinants of catastrophic healthcare expenditure in Peru," International Journal of Health Economics and Management, Springer, vol. 18(4), pages 425-436, December.

- Rezaei, S., Hajizadeh, M., Ahmadi, S., Sedghi, S., Piroozi, B., Mohamadi-Bolbanabad, A. and Homaie Rad, E. (2019), "Socioeconomic inequality in catastrophic healthcare expenditures in Western Iran: A decomposition analysis", International Journal of Social Economics, Vol. 46 No. 9, pp. 1049-1060. https://doi.org/10.1108/IJSE-01-2019-0034

- Tadiwos, Y.B., Kassahun, M.M. & Mebratie, A.D. Catastrophic and impoverishing out-of-pocket health expenditure in Ethiopia: evidence from the Ethiopia socioeconomic survey. Health Econ Rev 15, 15 (2025). https://doi.org/10.1186/s13561-025-00602-1

- Rezaei S, Hajizadeh M. Measuring and decomposing socioeconomic inequality in catastrophic healthcare expenditures in Iran. J Prev Med Public Health. 2019 Jul;52(4):214-223. doi: 10.3961/jpmph.19.046. Epub 2019 Jun 14. PMID: 31390684; PMCID: PMC6686108.

6- As the authors may know, there is a direct link between the prevalence of CHE and the methods of healthcare financing in a country. However, I did not find any information about the healthcare financing methods in the country or the percentage of total healthcare financing allocated to each method. Please add this information in the introduction section to provide international readers with better context.

7- Page 6: Please provide the exact number of households included in each wave, as well as the total number. I did not find this information in the current version of the paper.

8- Page 7: Please provide detailed information on how CHE was calculated in the study. Was there any adjustment for household size? How was CHE calculated if the cost of food for a household was greater than the subsistence costs? Please clarify these points and provide a more comprehensive explanation.

9- I spent a considerable amount of time trying to determine which assets were included in the PCA, but unfortunately, I could not find this information in the paper. Please revise the methods section to clearly specify which assets were included in the PCA.

10- In the study, the authors used only the 40% threshold of the household CTP to calculate the prevalence of CHE. Please conduct a sensitivity analysis by calculating the prevalence of CHE at the 10%, 20%, 30%, and 40% thresholds of capacity to pay (CTP), as recommended by previous studies.

11- As mentioned in my previous comments, the study title refers to socioeconomic inequalities, but the methods section does not explain how socioeconomic-related inequality in the prevalence of CHE was measured among the study population. Please use the references mentioned above to calculate the concentration index and concentration curve for the prevalence of CHE, and compare these measures over time. You can use the following reference to compute the concentration index (CI) and concentration curve (CC) for the prevalence of CHE using Stata software.

 O'Donnell O, O'Neill S, Van Ourti T, Walsh B. conindex: Estimation of concentration indices. Stata J. 2016 1st Quarter;16(1):112-138. PMID: 27053927; PMCID: PMC4819995.

12- The Results section is well written. No comments or issues caught my attention in this section.

13- The Discussion section needs to be rewritten to focus more closely on the study findings and to compare them with studies conducted in both developed and developing countries. Please use the following references to enrich the discussion and provide a broader context.

 Catastrophic health expenditure during the COVID-19 pandemic in five countries: a time-series analysis A. Haakenstad, C. Bintz, M. Knight, K. Bienhoff, H. Chacon-Torrico, W. H. Curioso, et al. The Lancet Global Health 2023 Vol. 11 Issue 10 Pages e1629-e1639 DOI: 10.1016/S2214-109X(23)00330-

? Al-Hanawi, M.K., Njagi, P. Assessing the inequality in out-of-pocket health expenditure among the chronically and non-chronically ill in Saudi Arabia: a Blinder-Oaxaca decomposition analysis. Int J Equity Health 21, 192 (2022). https://doi.org/10.1186/s12939-022-01810-5

? Rezaei S, Woldemichael A, Ebrahimi M, Ahmadi S. Trend and status of out-of-pocket payments for healthcare in Iran: equity and catastrophic effect. J Egypt Public Health Assoc. 2020 Nov 3;95(1):29. doi: 10.1186/s42506-020-00055-w. PMID: 33140214; PMCID: PMC7606373.

? Diego Proaño Falconi & Eduardo Bernabé, 2018. "Determinants of catastrophic healthcare expenditure in Peru," International Journal of Health Economics and Management, Springer, vol. 18(4), pages 425-436, December.

? Rezaei, S., Hajizadeh, M., Ahmadi, S., Sedghi, S., Piroozi, B., Mohamadi-Bolbanabad, A. and Homaie Rad, E. (2019), "Socioeconomic inequality in catastrophic healthcare expenditures in Western Iran: A decomposition analysis", International Journal of Social Economics, Vol. 46 No. 9, pp. 1049-1060. https://doi.org/10.1108/IJSE-01-2019-0034

? Tadiwos, Y.B., Kassahun, M.M. & Mebratie, A.D. Catastrophic and impoverishing out-of-pocket health expenditure in Ethiopia: evidence from the Ethiopia socioeconomic survey. Health Econ Rev 15, 15 (2025). https://doi.org/10.1186/s13561-025-00602-1

? Rezaei S, Hajizadeh M. Measuring and decomposing socioeconomic inequality in catastrophic healthcare expenditures in Iran. J Prev Med Public Health. 2019 Jul;52(4):214-223. doi: 10.3961/jpmph.19.046. Epub 2019 Jun 14. PMID: 31390684; PMCID: PMC6686108.

14- The study limitations section needs improvement. Please add that the study did not include important factors such as the age of household members, sex of the head of household, education level of the head of household, number of children in the household, presence of a person aged 60 years or older, and similar variables.

15- Policy implication is missing.

VERSION 1 - AUTHOR RESPONSE

Reviewer: 1

- **Abstract: The methods section should include all statistical methods used. For instance, how was mean and median values estimated. Once these estimates are accompanied by 95% CI it is no longer in the domain of descriptive statistics. This should be elaborated in the main methods as well.**
  We have now described what was included as descriptive statistics. We think how they were estimated is not commonly presented in scientific journals.

  Descriptive statistics (means and medians) and log ... (Page 1).

- **Data and Statistical Analysis: The authors said they used a before and after cross-sectional design, in which data from 13,581 and 28,970 participants were collected, respectively. Is the data paired or unpaired (parametric or non-parametric)? For this kind of impact analysis, paired data will be ideal to evaluate changes attributable to the policy, but the sample in each group suggests unpaired data was used. Could the authors clarify that?**

  Yes, the data were not paired as the samples in the two periods included randomly selected households. (Page 5).

  In each wave, the sampling method was multi-stage, stratified, and proportional to the size of the population. The households were randomly selected from a complete and updated list of occupied households, with the same probability of selection being applied to each house.

- **Having transformed the data on a log scale, did they check the assumption of normality and equal variance between groups before using the log-binomial regression? Any evidence of this assumption test in supplementary material will be helpful, as failure to check the assumption may lead to an inaccurate conclusion.**

  The data were not on a log scale. As mentioned in the manuscript, the regression model used a log-binomial distribution in order to estimate the prevalence ratio.

- **What statistical method did they use to calculate differences in the mean OOP between groups?**

  In this manuscript we have not calculated differences in mean OOP between groups. Instead, we present only descriptive statistics on OOP (in Table 1 and Figure 1).

- **Paired or unpaired t-test? Why present a median estimate? What statistical assumption informed that? Is the data parametric or non-parametric?**

  As mentioned in the previous comment, means and median were presented for descriptive purposes. Medians were included because of the asymmetric distribution of the expenditures.

- **Why use CPI to adjust costs from local currency to USD? The standard practice is to adjust using Purchasing Power Parity equivalent in USD for comparison of cost across countries. https://eppi.ioe.ac.uk/costconversion/**

  We did not adjust costs from local currency to USD because USD is the official currency in Ecuador. This we mention in the manuscript (Page 6): *"All expenditures were measured in US dollars ($), the current currency in Ecuador."* A concern was inflation and this adjustment we explain in the manuscript (Page 6): *"To facilitate comparison over time, the 2006 values were adjusted using the 2014 Consumer Price Index, multiplied by a factor of 1.41."*

- **Though 40% and 20% CHE thresholds are accurate, they could consider 15% and 25% standard thresholds. This will help understand the dynamics better as 40% is too high and may exclude several households and individuals who may experience impoverishment beyond, for example, at 15% threshold. Additional analysis showing catastrophic overshoot and impoverishment could have been more interesting as that speak volume to policymakers.**

We appreciate the reviewer's suggestions to add more analyses and note that the other reviewer suggests 10% and 30% thresholds. While we are fully aware of the practice to report results with several thresholds in previous studies, we believe these additional analyses have less value considering the focus of our manuscript on socioeconomic inequalities rather than only on the prevalence, as is more common in those studies that include both CHE and impoverishment in the same paper. It is for this reason that we report in the annex the results with the 20% CHE threshold and consider it sufficient to state that the pattern of socioeconomic inequalities remained consistent. Additionally, these are the common cut-offs in the Latin-American literature *(For instance, Amaya-Lara, J.L. Catastrophic expenditure due to out-of-pocket health payments and its determinants in Colombian households. Int J Equity Health. 2016; 15, 182.)*

- **Results: Some confidence intervals accompanying the point estimate are inaccurate, as the margin of error is inconsistent when checked using the lower and upper uncertainty intervals. The authors should check this error in all Tables containing 95% CI.**

  Thanks for the comment. We revised all analysis again without finding any errors. While symmetry is a common characteristic of many confidence intervals, it's not a universal rule, and the specific distribution of the estimator and the method used to calculate the interval determine whether it will be symmetric or not. For instance, when dealing with proportions, as in this case, confidence intervals are usually asymmetric.

- **Tables 2 and 1A: The presentation of wealth quintile groups may be misleading if the authors say Q1 is richest instead of poorest.**

  We have now clarified it in the methods section. (Page 6 and tables).

  ... and the index was divided into quintiles, with the first quintile as the richest.

Reviewer: 2

- **First of all, please revise the study title to better reflect the objectives. The current title is vague and misleading. I believe that a before-and-after cross-sectional study design is unnecessary.**

  Thank you, we have changed the title according to this comment. (Page 1):

  Did health reform improve financial protection for disadvantaged groups in Ecuador? A socio-economic inequality assessment of catastrophic health expenditures 2006-2014

- **In the study title, the phrase "health reform" appears, yet there is no information about health reforms in the abstract. Please either add more details about health reforms in the abstract or consider changing the study title accordingly.**

  Thank you for your comment. We have clarified the abstract's introduction. (Page 1).

  Latin American countries have long struggled with socioeconomic inequalities in health. In 2007, Ecuador implemented a health reform to address these issues, including by making public health services free, coordinating finances between subsystems, and increasing the state's

health budget. This study assessed whether Ecuador's health system reform (2007-2017) reduced out-of-pocket health spending (OOP), catastrophic health spending (CHS), and socioeconomic inequalities in CHS.

- **Page 3, STRENGTHS AND LIMITATIONS OF THIS STUDY: The authors stated, "The method for calculating CHE could limit comparisons between countries in the region and globally." I could not understand this limitation clearly. Please revise this statement for clarity or consider deleting it.**

  Thank you, this statement has been deleted to avoid confusion.

- **Page 3, the authors stated, "Although the healthcare reform was implemented between 2007 and 2017, the study only included data from two points in time (2006 and 2014), which are available at the moment." It appears that the study did not compare the prevalence of CHE before and after the health reforms. Therefore, I suggest rephrasing or removing references to health reform throughout the paper, including the study title.**
  The health reform initiated in 2007 serves as the basis for this study. We agree with the reviewer´s comment and we have adjusted the text for clarity. Additionally, we have deleted the description of the study design as a "before-and-after study". (Page 5).

  This was a cross-sectional study that used publicly available secondary data from the National Living Standards Measurement surveys. These were conducted by the National Institute of Statistics and Censuses (Instituto Nacional de Estadísticas y Censos in Spanish) in 2006 (pre-reform) and 2014 (during the reform) across the country.

- **The introduction should be rewritten to focus more on studies conducted in other countries. Please incorporate the following references in both the introduction and discussion sections to enrich and improve the paper.**

  Most references pertain to the regional context, including works like Diego Proaño Falconi & Eduardo Bernabé's 2018 study, "Determinants of Catastrophic Healthcare Expenditure in Peru," which is already cited in the manuscript. However, we´ve rewritten some paragraphs for clarity, also we have added following references in the discussion section.

  - *Rezaei S, Woldemichael A, Ebrahimi M, Ahmadi S. Trend and status of out-of-pocket payments for healthcare in Iran: equity and catastrophic effect. J Egypt Public Health Assoc. 2020 Nov 3;95(1):29.*
  - *Tadiwos, Y.B., Kassahun, M.M. & Mebratie, A.D. Catastrophic and impoverishing out-of-pocket health expenditure in Ethiopia: evidence from the Ethiopia socioeconomic survey. Health Econ Rev 15, 15 (2025).*
  - *Rezaei S, Hajizadeh M. Measuring and decomposing socioeconomic inequality in catastrophic healthcare expenditures in Iran. J Prev Med Public Health. 2019 Jul;52(4):214-223.*

- **As the authors may know, there is a direct link between the prevalence of CHE and the methods of healthcare financing in a country. However, I did not find any information about the healthcare financing methods in the country, or the percentage of total healthcare financing allocated to each method. Please add this information in the introduction section to provide international readers with better context.**

Thanks for the comment. In the Methods section, we have detailed the financing model and the allocation of the health budget investment. However, we have now added some sentences to include healthcare financing. (Pages 3-4).

The remaining 10% of the population was covered by the private healthcare sector. The MoPH receives financial resources from the Ministry of Finance based on historical assignments and mainly from taxes, oil revenues and external funds. Subsequently, the funds are distributed to administrative units and hospital health facilities throughout the country. The social security systems are financed through compulsory payroll taxes and voluntary user contributions, with the provision of services conducted through the utilisation of their own facilities. The MoPH accounts for over half of the total health expenses in the public system. In the private sector, health insurance companies operate prepaid drug schemes financed by beneficiary contributions. During this period, approximately 27% of health needs remained unmet due to limited access to services

- **Page 6: Please provide the exact number of households included in each wave, as well as the total number. I did not find this information in the current version of the paper.**
  On page 5, the methods section states:

  The surveys provided nationally representative data from 13,581 and 28,970 households in each wave respectively.

- **Page 7: Please provide detailed information on how CHE was calculated in the study. Was there any adjustment for household size? How was CHE calculated if the cost of food for a household was greater than the subsistence costs? Please clarify these points and provide a more comprehensive explanation.**

Page 5-6 details the calculation of the CHE. The cost of food is equivalent to subsistence costs, and we explained that the CHE arise when it exceeds the capacity to pay (CTP), which encompasses non-food expenditures.

The CHE was constructed in two steps. First, the household's capacity to pay (CTP) was calculated. CTP was defined as the household's non-food expenditure in a given period and included four types of expenditure: (i) expenditure on fuel and energy in the last week; (ii) expenditure on household and personal care products, entertainment, culture, membership fees and domestic services in the last month; (iii) expenditure on clothing and footwear in the last three months; and (iv) expenditure on appliances, furniture, leisure, sports equipment, jewellery, vehicles, transport, professional services, prepaid fees, taxes and transfers to family members in the last year. Second, CHE was determined using the methodology proposed by WHO in 2004, where households were exposed to CHE if their out-of-pocket health expenditure was equal to or greater than 40% of their CTP.[27 28] This approach allowed us to compare our findings with data from previous studies conducted nationally and from neighbouring countries in the region.

In addition, due to the structure of the datasets, it was not possible to use information about household size in the analysis. We have added this as a limitation. (Page 2).
Due to the structure of the datasets, it was not possible to use other relevant data in this analysis such as household size, the age of the household members and the education level of the household head.

- **I spent a considerable amount of time trying to determine which assets were included in the PCA, but unfortunately, I could not find this information in the paper. Please revise the methods section to clearly specify which assets were included in the PCA.**
  Thanks. We have now included this information. (Page 6).

  A household wealth index was derived using principal component analysis of 15 household assets reported in the survey such as doorway materials, roof, wall and floor material of household, household construction, cooking facility, cooking fuel, toilet, water source, lighting source, landline telephone, internet at home, satellite TV and household waste disposal. The PCA was run separately for 2006 and 2014, and the index was divided into quintiles, with the first quintile as the richest.

- **In the study, the authors used only the 40% threshold of the household CTP to calculate the prevalence of CHE. Please conduct a sensitivity analysis by calculating the prevalence of CHE at the 10%, 20%, 30%, and 40% thresholds of capacity to pay (CTP), as recommended by previous studies.**

  We report in the annex the results with the 20% CHE threshold. We appreciate the reviewer's suggestions to add sensitivity analyses and note that the other reviewer suggests 15% and 25% thresholds. While we are fully aware of the practice to report results with several thresholds in previous studies on CHE prevalence, we believe these additional analyses have less value in our manuscript because the focus of our manuscript is on socioeconomic inequalities rather than only on the prevalence. It is for this reason that we consider it sufficient to report the results with 20% in the Annex and comment in the manuscript that the pattern of socioeconomic inequalities remained consistent. Additionally, 10% is rarely used in the regional context whereas 20% and 40% are the common cut-offs in the Latin-American literature *(For instance, Amaya-Lara, J.L. Catastrophic expenditure due to out-of-pocket health payments and its determinants in Colombian households. Int J Equity Health. 2016; 15, 182.)*

- **As mentioned in my previous comments, the study title refers to socioeconomic inequalities, but the methods section does not explain how socioeconomic-related inequality in the prevalence of CHE was measured among the study population. Please use the references mentioned above to calculate the concentration index and concentration curve for the prevalence of CHE and compare these measures over time. You can use the following reference to compute the concentration index (CI) and concentration curve (CC) for the prevalence of CHE using Stata software. § O'Donnell O, O'Neill S, Van Ourti T, Walsh B. conindex: Estimation of concentration indices. Stata J. 2016 1st Quarter;16(1):112-138. PMID: 27053927; PMCID: PMC4819995**.

  Thanks for the comment. We have now provided a clearer description in the manuscript (Page 7).

  For the second objective, a log-binomial regression model was used to calculate the relative risk of CHE for each socio-economic variable in 2006 and 2014. Thus, socio-economic inequalities were measured as differences between groups using 95% confidence intervals (CI) for statistical inference. First, a univariable model (model 1) was estimated, followed by a multivariable model (model 2) including all statistically significant variables from model 1. Finally, an interaction term was added to assess relative risk changes over time, expressed as relative differences (RD). RD less than one implies a reduction in relative risk over time compared to the reference group and thus a decrease in inequality.

While we are aware that concentration index is another measure of health inequality, it did not fit well to be applied to the research question and data of this study. Specifically, our aim was to assess CHE inequality along different socio-economic dimensions of which only wealth was a continuous variable. Thus, it was appropriate to use relative risk and to assess whether relative risk changes over time in the different socio-economic groups.

- **The Results section is well written. No comments or issues caught my attention in this section.**

  Thanks for this comment.

- **The Discussion section needs to be rewritten to focus more closely on the study findings and to compare them with studies conducted in both developed and developing countries. Please use the following references to enrich the discussion and provide a broader context.**

  Thanks, we have added some references that used similar methodologies to assess the CHE.

  - *Rezaei S, Woldemichael A, Ebrahimi M, Ahmadi S. Trend and status of out-of-pocket payments for healthcare in Iran: equity and catastrophic effect. J Egypt Public Health Assoc. 2020 Nov 3;95(1):29.*
  - *Tadiwos, Y.B., Kassahun, M.M. & Mebratie, A.D. Catastrophic and impoverishing out-of-pocket health expenditure in Ethiopia: evidence from the Ethiopia socioeconomic survey. Health Econ Rev 15, 15 (2025).*
  - *Rezaei S, Hajizadeh M. Measuring and decomposing socioeconomic inequality in catastrophic healthcare expenditures in Iran. J Prev Med Public Health. 2019 Jul;52(4):214-223.*

  We have also summarised the study findings in the first paragraph of the Discussion more in detail, to better prepare for the following paragraphs, as follows: (Page 8).
  This study evaluated the impact of the recent reform of the Ecuadorian health system on improving financial protection in health care. Our results showed a reduction in households' direct health care expenditure and CHE during the reform compared to the pre-reform period. A significant reduction in socio-economic inequalities in CHE was also observed between the richest and the poorest and between urban and rural households over time. However, inequalities in CHE increased for the Andean region and for uninsured households.

- **The study limitations section needs improvement. Please add that the study did not include important factors such as the age of household members, sex of the head of household, education level of the head of household, number of children in the household, presence of a person aged 60 years or older, and similar variables.**
  Thanks, we've added this to the limitations bullet points. (Page 2).

  Due to the structure of the datasets, it was not possible to use other relevant data in this analysis such as household size, the age of the household members and the education level of the household head.

- **Policy implication is missing.**

We have revised the conclusion section including the policy implications. (Page 9).

Conclusion

The recent reform in Ecuador has reduced both total out-of-pocket expenditure and catastrophic health expenditure. It has also improved financial protection for the most disadvantaged groups, notably reducing socio-economic inequalities in CHE over time, except in the Andean region and among uninsured households. Therefore, it is imperative to review and adapt the reforms implemented to expand and sustain universal access to health care and improve health insurance coverage for the most disadvantaged groups. A forward-looking approach to the financial sustainability of the public health care model is required to ensure the right to health in the country.

VERSION 2 - REVIEW

Reviewer 1

Name Otieku, Evans

Affiliation University of Ghana, Institute of Statistical, Social and Economic Research

Date 12-Jul-2025

COI

No further comments to the authors. Their response to my first review comments is satisfactory.

Reviewer 2

Name Rezaei, Satar

Affiliation Kermanshah University of Medical Sciences, School of Public Health

Date 11-Jul-2025

COI

All of my comments addressed by the authors in the revised version of the paper and now it is ready to publication in the journal.
